# Expert opinion on translational research for advanced glioblastoma treatment

**DOI:** 10.20892/j.issn.2095-3941.2023.0012

**Published:** 2023-04-25

**Authors:** Xiaoteng Cui, Yunfei Wang, Junhu Zhou, Qixue Wang, Chunsheng Kang

**Affiliations:** Laboratory of Neuro-oncology, Tianjin Neurological Institute, Key Laboratory of Post-Neuro Injury Neuro-Repair and Regeneration in Central Nervous System, Ministry of Education and Tianjin City, Tianjin Medical University General Hospital, Tianjin 300052, China

**Keywords:** Malignant gliomas, glioblastoma, temozolomide, chemoresistance, small molecule drugs

## Abstract

Malignant gliomas are known to be one of the most difficult diseases to diagnose and treat because of the infiltrative growth pattern, rapid progression, and poor prognosis. Many antitumor drugs are not ideal for the treatment of gliomas due to the blood-brain barrier. Temozolomide (TMZ) is a DNA alkylating agent that can cross the blood-brain barrier. As the only first-line chemotherapeutic drug for malignant gliomas at present, TMZ is widely utilized to provide a survival benefit; however, some patients are inherently insensitive to TMZ. In addition, patients could develop acquired resistance during TMZ treatment, which limits antitumor efficacy. To clarify the mechanism underlying TMZ resistance, numerous studies have provided multilevel solutions, such as improving the effective concentration of TMZ in tumors and developing novel small molecule drugs. This review discusses the in-depth mechanisms underlying TMZ drug resistance, thus aiming to provide possibilities for the establishment of personalized therapeutic strategies against malignant gliomas and the accelerated development and transformation of new targeted drugs.

## Introduction

A malignant glioma is the most common primary tumor originating in the central nervous system (CNS)^1,2^. According to the 5^th^ edition of the World Health Organization (WHO) classification of CNS tumors, gliomas are categorized as grades 1–4^3^. WHO grade 4 isocitrate dehydrogenase-wild type (IDH-WT) gliomas are also known as glioblastomas (GBMs) and generally have a median survival time of 12–15 months after diagnosis^4^. Because GBMs present with malignant behavior (i.e., invasive growth) into normal brain tissue, complete resection is nearly impossible^2^. The vast majority of GBMs recur within a few months after surgery, even if the residual tumor is not detected on magnetic resonance imaging (MRI) after extensive resection^5^. Recurrent GBMs progress rapidly without appropriate treatment and causes significant pain; however, use of alkylating agents as chemotherapy drugs in patients with GBMs has significantly improved survival^6^. Indeed, maximal surgical resection, followed by radiotherapy and alkylating agent chemotherapy, has become the conventional therapeutic strategy for patients with GBM^7,8^.

Temozolomide (TMZ), an oral alkylating agent, was first discovered to have antitumor effects in 1987^9^ and was approved as a conventional frontline chemotherapeutic agent for GBM treatment by the FDA in 2005^10^. TMZ has the following advantages: high efficiency in crossing the blood-brain barrier (BBB); maintaining a high drug concentration within the GBMs; and exerting antitumor effects^11,12^. Although TMZ treatment has improved the survival time and quality of life of GBM patients, TMZ is still considered to be palliative treatment^13,14^. With the widespread use of TMZ, the emerging problem of drug resistance has led to GBM treatment failure^11^. In recent years, physical^15^, chemical^16,17^, and biological^18^ treatment strategies have been applied in clinical trials or clinical therapies involving GBMs, but the clinical efficacies have not proven satisfactory. Thus, there is an urgent need to establish a reasonable GBM treatment strategy to effectively prolong patient survival and quality of life.

## TMZ is currently the optimal and only available chemotherapeutic drug for GBM treatment

### Cytotoxic mechanism of TMZ action

**Figure 1:**
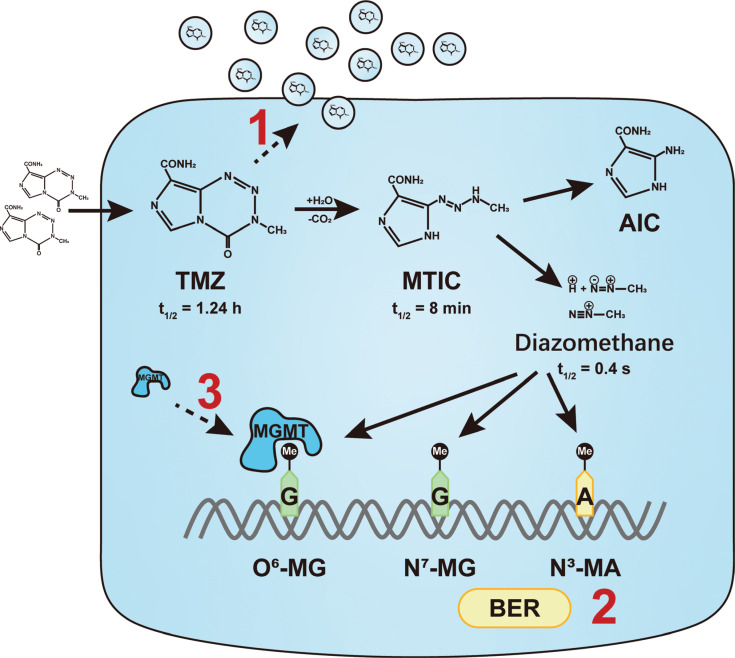
Intracellular metabolism and drug resistance mechanisms of Temozolomide (TMZ). TMZ, an alkylating agent that can cross the BBB, is hydrolyzed and metabolized into the active ingredient, diazomethane. Diazomethane has a very short half-life in cells and methylates guanine and adenine of DNA to form N^7^-MG, O^6^-MG, and N^3^-MA. A considerable portion of TMZ, however, is secreted from the cell in the form of the original drug after entering cells. In addition, the activation of the base excision repair (BER) mechanism and the high level of O^6^-methylguanine-DNA methyltransferase (MGMT) rapidly erases cytotoxic methylated modifications, resulting in cell survival. MTIC: 5-(3-methyltriazen-1-yl) imidazole-4-carboxamide; AIC: 4-amino-5-imidazole-carboxamide.

TMZ is the first-line drug for clinical GBM therapy. TMZ is a lipophilic molecule with oral administration and is well-tolerated. Pharmacokinetic studies have shown that TMZ is rapidly hydrolyzed to the 5-(3-methyltriazen-1-yl) imidazole-4-carboxamide (MTIC) metabolite within 1.5 h after entering the cell. MTIC is further metabolized to produce the active ingredient, diazomethane. The MTIC half-life is 8 min, which causes methylation of the guanine residues, O^6^ and N^7^, and the adenine residue, N^3^, during DNA replication, thus forming O^6^-MG, N^7^-MG, and N^3^-MA, respectively^19,20^. These abnormal modifications cause DNA strand breaks and replication fork collapse, which leads to cell cycle arrest in the G2/M phase and ultimately contributes to apoptosis^21–23^ (**Figure 1**).

### Mechanism underlying TMZ resistance

#### Deficiency of the effective TMZ intracellular concentration

The effective concentration of a chemotherapeutic drug at the tumor site is a crucial factor in determining efficacy. TMZ is highly enriched in gliomas due to the efficient ability to cross the BBB, which is an important feature that distinguishes TMZ from other alkylating agents. Tumor cells actively secrete intracellular chemotherapy drugs, which leads to insufficient concentrations of drugs, and thus the cytotoxic effects of drugs are diminished. For example, P-glycoprotein (P-gp), which is encoded by the multidrug resistance protein 1 (MDR1) gene, is a drug efflux pump involved in chemotherapy resistance that is overexpressed on the surface of tumor cells^24,25^. P-gp has been reported to be expressed in most brain tumors, including GBM, and P-gp may be involved in TMZ resistance^26,27^. In addition, our study demonstrated that the exocrine function of GBM is a vital mechanism underlying primary resistance against TMZ. After entering the cells, nearly one-half of TMZ is secreted through the vesicle system in the form of the original drug^28^. All of the above-cited reports suggest that the reduction in the effective concentration of TMZ in tumors caused by various mechanisms is the first step leading to chemotherapy resistance.

#### Abnormal DNA damage repair (DDR) function

DDR is a fundamental cellular function to compensate for DNA lesions. The O^6^-MG caused by TMZ changes the normal hydrogen bond between guanine and cytosine, which leads to mismatches with thymine during DNA replication, then triggers cellular repair *via* the mismatch repair (MMR) mechanism. MMR recognizes and excises mispairing in the new strand but leaves O^6^-MG in the parent strand, which results in futile cycle repeats and eventually leads to cell death^29^. MutS homolog 6 (MSH6), which is a member of the MutS family of proteins involved in MMR, is commonly mutated in patients with recurrent GBM after receiving TMZ chemotherapy when compared to patients with primary GBM^30,31^. This finding indicates that key gene mutations lead to abnormal MMR function, thus causing TMZ resistance^32^.

Base excision repair (BER) is another essential DNA repair mechanism. After exposure to chemical drugs or ionizing radiation, damaged DNA can rapidly activate damage recognition proteins and repair damaged DNA fragments through a series of cutting, joining, and other processes with a series of enzymatic reactions^33^. N^7^-MG and N^3^-MA activate poly (ADP-ribose) polymerase 1 (PARP-1), promote X-ray repair cross complementing 1 (XRCC1), repair enzyme recruitment at DNA damage sites, and activate the BER repair process, resulting in cell survival^34^. Furthermore, PARP inhibitors sensitize glioma cells to TMZ^35^.

#### O6-methylguanine-DNA methyltransferase (MGMT)-mediated specific repair

Given that N^3^-MA and N^7^-MG are rapidly removed by BER before DNA replication, O^6^-MG is the dominant position at which TMZ causes DNA damage. MGMT is a DNA repair enzyme that removes methylation of the O^6^ site on DNA, thus repairing the cytotoxic damage caused by TMZ^11,36^. This effect is rapid and specific, so the level of MGMT expression is thought to reflect the clinical efficacy of TMZ in patients with gliomas.

Numerous studies have shown that MGMT expression is mainly regulated by the promoter region methylation level^37,38^. Epigenetic silencing of MGMT by promoter hypermethylation is significantly associated with longer survival in glioma patients who receive TMZ chemotherapy^39^. Intense MGMT histochemical staining in glioma samples is associated with TMZ resistance^40^. It has been reported that long non-coding RNAs (lncRNAs), such as lnc-TALC and HOTAIR, regulate MGMT expression *via* epigenetic modification alterations^41,42^. These findings have helped neurosurgeons judge the effectiveness of TMZ treatment and the survival time of GBM patients by evaluating the MGMT gene promoter methylation level in tumor tissues. Moreover, clinical data have suggested that TMZ treatment also helps prolong the survival time of GBM patients with an unmethylated MGMT promoter^39,43^. Therefore, regardless of the MGMT promoter status, TMZ is currently the only chemotherapeutic drug used for glioma patients after surgery.

## Strategies for better therapy against GBM

Currently, potential strategies for TMZ resistance are shown in **Table 1**. To solve the problem of GBM treatment efficacy, it is important to fully explore the pathogenesis and chemoresistance mechanisms underlying GBM, establish novel diagnosis and treatment strategies, and develop new therapeutic approaches.

**Table 1:** Mechanisms of TMZ resistance and potential therapeutic strategies

Mechanisms of TMZ resistance	Markers or regulatory pathways	Potential therapeutic strategies
Insufficient intracellular concentration of TMZ		
Extruded through an efflux pump	P-gp encoded MDR1^26,27^	Tariquidar^44^
Extruded through a vesicle system	PTRF/Cavin-1	Sequential therapy of TMZ followed by chloroquine^28^
Aberrant DDR		
MMR inactivation	MSH6 mutation^30,31^	
BER hyperactivation	PARP-1/XRCC1 axis^35^	Olaparib^45^
Specific repair by MGMT		
MGMT PARylation	PARP-mediated PARylation	Olaparib^34^
Epigenetic regulation of MGMT	ATF3/p-p65/HADC1 axis	EPIC-0412^42^

### Novel diagnostic and therapeutic schemes based on deep sequencing technology

Gene mutations cause tumor occurrences and are key factors affecting the clinical therapeutic effect. In recent years studies have shown that GBMs have a variety of somatic mutations with subtype specificities^46^. The molecular subtypes of GBMs can be determined by genome and transcriptome sequencing^47^. The typical features of the GBM-classical subtype are chromosome 7 amplifications accompanied by chromosome 10 deletions. These mutations are characterized by the high expression of epidermal growth factor receptor (EGFR) and deletion of cyclin-dependent kinase inhibitor 2A (CDKN2A), but the TP53 gene is generally not mutated in this subtype. The neuronal precursor cell and stem cell marker gene, Nestin, as well as Notch and Hedgehog pathway-related genes, are highly expressed in this subtype. The most common genomic alterations of the mesenchymal subtype of GBM are deletions of neurofibromin 1 (NF1) and phosphatase and tensin homolog (PTEN), which lead to the continuous activation of the protein kinase B (PKB, also known as AKT) pathway. The characteristic highly-expressed genes of this subtype are mesenchymal-related genes, such as chitinase 3-like 1 (CHI3L1), MET, CD44, and MERTK, and tumor necrosis factor alpha (TNF-α) and nuclear factor κB (NF-κB) pathway-related genes. The GBM-proneural subtype is characterized by somatic mutations of IDH1 and platelet-derived growth factor receptor alpha (PDGFRA), which are often accompanied by TP53 deletions. The oligodendrocyte development-related gene oligodendrocyte transcription factor 2 (OLIG2), NK2 homeobox 2 (NKX2-2), and the SOX protein family are highly expressed in this subtype. The significant features of the neural subtype of GBM are the high levels of neuron marker gene expression, such as neurofilament light chain (NEFL), gamma-aminobutyric acid type A receptor subunit alpha 1 (GABRA1), synaptotagmin 1 (SYT1), and solute carrier family 12 member 5 (SLC12A5).

Understanding the characteristics of gene mutations in tumors can be helpful to evaluate the therapeutic strategies and prognostic conditions for tumor patients. IDH1/2 mutations are mostly found in low-grade gliomas and secondary GBMs that have a good prognosis for radiotherapy and alkylating agent chemotherapy^48^. Genetic mutations of the telomerase reverse transcriptase (TERT) promoter region do not co-exist with IDH1 mutations in primary GBMs; however, co-mutations in TERT promoter and IDH1/2 are often detected in oligodendrogliomas, which indicates a good prognosis^49^. For GBM patients with the V600E mutation in the B-Raf proto-oncogene, serine/threonine kinase (BRAF) gene, the BRAF inhibitor, vemurafenib is recommended for adjuvant treatment^50^. Hypermethylation of the MGMT promoter region indicates that TMZ chemotherapy would be more beneficial for GBM patients^51^.

With the rapid development of sequencing technology, more GBM-specific mutations and low-frequency loci will be gradually revealed and studied. Omics studies have shown that adult diffuse gliomas in East Asians have unique genomic characteristics^52^. By further promoting the genomic diagnosis of GBM in the clinical setting, researchers can make personalized diagnoses at the molecular level and guide the establishment of individual therapeutic strategies. Deep sequencing analysis of large samples allows for the thorough analysis of the effects of different genetic mutations on the progression and prognosis of GBMs. Moreover, under the orchestration of deep sequencing and multiomics analysis, novel marker diagnostic kits can be developed for the better diagnosis of GBMs and the implication of precise treatments against malignant GBMs. Based on the above findings, the possibilities of gene mutations as new therapeutic targets are worth evaluating for the development of targeted small molecule drugs. Additionally, these mutations actively promote clinical transformation, provide a molecular theoretical basis for effective treatment approaches for personalized diagnosis and treatment, and eventually improve the prognosis of GBM patients.

### New drugs and new technologic breakthroughs

Because of the special origination site and unique microenvironment characteristics of brain tumors, the majority of drugs are difficult to pass through the BBB, which greatly limits the therapeutic effect. With inherent high heterogeneity, gliomas often recur and develop treatment resistance. In recent years, cancer immunotherapies using chimeric antigen receptor T (CAR-T) cells have been adopted in clinical treatments. CAR-T cells show strong antitumor effects in hematologic malignancies, but challenges remain in the treatment of solid tumors, including malignant gliomas^53^. Immune checkpoint inhibitors significantly benefit patients with solid tumors, such as lung cancers or melanomas, but are not ideal for GBM patients^54–56^. With ongoing research, novel drugs targeting new targets have shown satisfactory effects against malignant gliomas, and some drugs have been approved for clinical trials. Such drugs include AG-120 and AG-881, which target IDH1 mutations^57,58^, bozitinib, which targets the PTPRZ1-MET fusion gene^16^, and ACT001, a Chinese medicinal extract from the root bark of magnolia that blocks the activity of the phosphatidylinositide 3-kinase (PI3K) pathway^59^. The clustered regularly spaced short palindromic repeats (CRISPR)/Cas13a system has also shown promising preclinical results in the treatment of GBM by virtue of its unique gene editing ability to target RNA^60^. With respect to new uses for old drugs, the widely used antidiabetic drug, metformin, inhibits GBM growth by altering the metabolic state and inducing apoptosis of tumor cells^61^. Sequential therapy of chloroquine and TMZ has shown good effects against GBMs, as the concentration of TMZ increased in tumor cells by the chloroquine-induced inhibition of exocrine function^28^. Numerous studies demonstrated that TMZ-resistant GBM cells transmit resistance *via* exosomes^62–65^. Targeting exosomes has great potential to reverse TMZ resistance. In addition, Lin and colleagues^66^ reported a novel analog of TMZ that induced MMR-independent cell killing selectively in TMZ-resistant tumors with MGMT silence.

At present, the clinical treatment of GBMs is still facing limitations. Novel therapeutic drugs and treatment approaches need to be developed. Advanced drug delivery systems would help to solve the crucial problems of cross-BBB delivery. The in-depth mechanisms of old drugs for GBMs and appropriate drug combinations are worth exploring. Moreover, accelerating the developments and clinical transformations of new small-molecule drugs provide a promising future.

### Deeper research focusing on the GBM microenvironment will help to develop novel therapeutic targets

GBMs are a kind of well-known “cold tumor” because of insensitivity to most current immunotherapies^67^. In recent years, the wide application of single-cell RNA sequencing technology in tumors has greatly improved the understanding of the complex tumor microenvironment and cell-cell interactions in GBMs. By analyzing the single-cell transcriptome data of clinical samples, the high inter- and intra-heterogeneity of GBMs were further revealed^68^, and malignant cells in GBM samples were sorted into four subtypes by means of the expression of signature genes^69^. Furthermore, it was found that a large number of macrophages/microglia infiltrated GBM tissues, whereas the proportion of T lymphocytes was very small^70^. An in-depth study showed that many bone marrow-derived macrophages exist in tumor tissues, while brain-resident microglia mostly appear around GBM tissues^71^. Moreover, single-cell sequencing analysis of immune cells sorted from GBM samples *via* CD45 or CD11b antibodies revealed the cell types, distribution, and transcriptomic features of immune cells in GBMs^70,71^. The above findings not only reveal the unique landscape of the immune microenvironment in GBMs but will also benefit the development of therapeutic targets based on cell-cell interactions. For example, colony stimulating factor 1 receptor (CSF1R), also known as M-CSF, is mainly expressed on the cell surface and is critical for macrophage differentiation and survival^72^. CSF1 is a secretory ligand of CSF1R that is secreted by tumor cells. CSF1 can bind and activate the tyrosine kinase activity of CSF1R to promote macrophage M2 polarization. Inhibitors blocking the interaction between CSF1/CSF1R reverses the immunosuppressive tumor microenvironment. For example, BLZ945, PLX3397, and other drugs targeting the CSF1/CSF1R interaction have displayed promising antitumor effects and have been used in clinical trials to treat tumors^73,74^. The results of preclinical experiments showed that PLX3397 has a good inhibitory effect against GBMs driven by platelet-derived growth factor subunit B (PDGFB); however, PLX3397 promotes RAS-driven GBM growth and has no effect on other mesenchymal or pre-neural subtypes^75^.

Cancer stem cells are a type of cell with self-renewal and multidirectional differentiation potential in tumor tissues. Cancer stem cells have a key role in maintaining tumor heterogeneity, promoting tumor progression, and driving tumor recurrence^76,77^. Studies have demonstrated that there are cells expressing CD133, SOX2, and other stem genes in GBM tissues, and these cells are defined as glioma stem cells (GSCs)^78^. It has been reported that GSCs not only have the abilities of self-renewal and multilineage differentiation but are also related to chemo- and radio-therapy resistance^79^. The hypoxic and ischemic microenvironment in GBMs promotes angiogenesis mediated by GSCs^80^ and accelerates the invasion and metastasis of GBMs^81^. Targeting GSCs would be expected to prolong the tumor-free survival time of GBM patients.

Overall, the unique immune microenvironment of GBMs requires more specific immune targets and immunotherapy strategies. The investigation of the in-depth mechanisms of tumorigenesis and the development of novel targeted drugs are worth pursuing to reverse the current status of GBMs as a “cold tumor”.

### Applications of new tumor animal models and novel experimental technologies that mimic clinical tumors for promoting preclinical mechanistic research on GBM

Appropriate experimental models can promote the rapid development of mechanistic explorations and drug discoveries. Based on the gene mutation characteristics of distinct GBM subtypes, scientists have established several subtype-specific GBM primary models^82^. In 2000, Holland and colleagues^83^ transfected the K-Ras (G12D) and AKT (Δ11–60) mutants into immortalized chicken fibroblasts and injected these cells into mouse brain. The treated cells showed glioma-like growth, forming infiltrating diffuse tumor tissue. Thus, activated Ras and AKT pathways induce diffuse glioma. In addition, a recombinant lentivirus with PDGFB overexpression and CDKN2A knockdown injected into the hippocampus of mice induced the occurrence of proneural subtype GBMs^84^.

The increasing development of 3D cell culture technology enables the realization of tumor organoids. The 3D system showed the complex spatial morphology of tumor tissues and the cell-cell and cell-matrix interactions and has unique applications in preclinical drug screening and evaluations. At present, many studies on glioma-like organoids have been carried out^85,86^.

3D printing has become a novel technology in recent years. Some studies have applied 3D biological printing technology to construct *ex vivo* models of GBM *via* the mixed bioprinting of various cells and matrices in petri dishes for culture and observation to obtain GBM models close to real tumors and explore the interactions between tumor and immune cells in the microenvironment^87^.

Natural and reasonable GBM models are very important for basic research and preclinical tests of novel drugs. Accelerating the development of animal models based on the genetic characteristics of diseases, establishing animal models that better represent clinical GBMs, and focusing on promoting organoid culture and 3D culture technology help to facilitate the rapid advancement of basic research and drug screening of GBMs.

## Conclusions

Gliomas have always been one of the most malignant diseases hindering human health. Researchers have made continuous efforts to explore the pathogenesis, microenvironment characteristics, and drug resistance mechanisms of this disease, revealing an increasing number of potential drug targets. Although TMZ is still the only drug available, we have reason to believe that the development of personalized diagnoses will provide more individualized treatment strategies for GBM patients.
